# The Extra Cusp Mystery: Exploring Dens Evaginatus

**DOI:** 10.7759/cureus.94526

**Published:** 2025-10-14

**Authors:** Rachid Boudi, Majid Sakout, Hind Ramdi

**Affiliations:** 1 Department of Pediatric Dentistry, Faculty of Dental Medicine, Mohammed V University in Rabat, Rabat, MAR; 2 Department of Conservative Dentistry and Endodontics, Faculty of Dentistry, Mohammed V Military Hospital, Mohammed V University in Rabat, Rabat, MAR

**Keywords:** dens evaginatus, occlusal tubercule, prophylactic approach, rare case, tooth anomaly

## Abstract

Dens evaginatus (DE) is a shape abnormality characterized by a tubercle extending over the surface of the affected tooth. This tubercle is usually found in the main grooves of the molars and premolars. It is vulnerable to fractures or wear that can cause inflammation. This may be followed, or not, by pulp necrosis due to the irritation of the pulp in the affected tubercle.

We present a case of dens evaginatus that did not involve pulpal extension, managed using a dual prophylactic strategy. This case report highlights the importance of prompt diagnosis and intervention.

## Introduction

Dens evaginatus (DE) is a rare developmental anomaly characterized by an additional cusp or tubercle protruding from the occlusal surface of the posterior teeth, most frequently the premolars. This cusp results from the abnormal proliferation of the inner enamel epithelium and dental papilla into the stellate reticulum during tooth development, leading to the formation of a supplemental elevation of enamel and dentin that may or may not contain pulpal tissue [1,2].

The condition is typically bilateral and occurs more frequently in the mandible than in the maxilla, with mandibular second premolars most commonly affected. Its reported prevalence ranges from 1% to 4%, with higher occurrence in Asian and Eskimo populations compared to Caucasian populations [1].

Clinically, dens evaginatus is significant because the tubercle is highly prone to occlusal interference, premature wear, or fracture during mastication. Such damage can expose dentin or pulp, resulting in pulpitis, pulpal necrosis, or periapical inflammation. Early recognition and preventive intervention are therefore essential to avoid complications and maintain tooth vitality [3].

This article presents a clinical case of bilateral dens evaginatus on mandibular premolars, emphasizing the importance of early detection and preventive management through conservative treatment.

## Case presentation

A 13-year-old Moroccan boy was referred to the Dental Treatment Consultation Center in Rabat for regular dental care. His medical history was normal, and there was no family history of supernumerary teeth or dens evaginatus.

**Figure 1:**
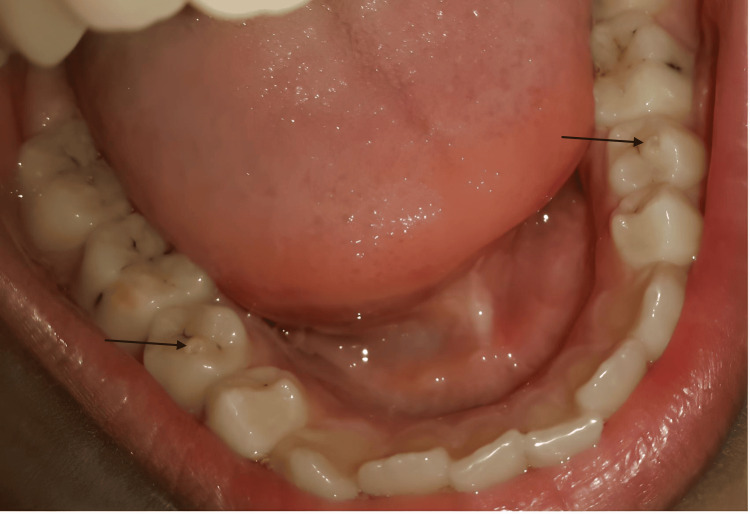
Occlusal view of mandibular teeth with dens evaginatus on both second premolars (black arrows) and MIH on the molars MIH: molar incisor hypomineralization

**Figure 2:**
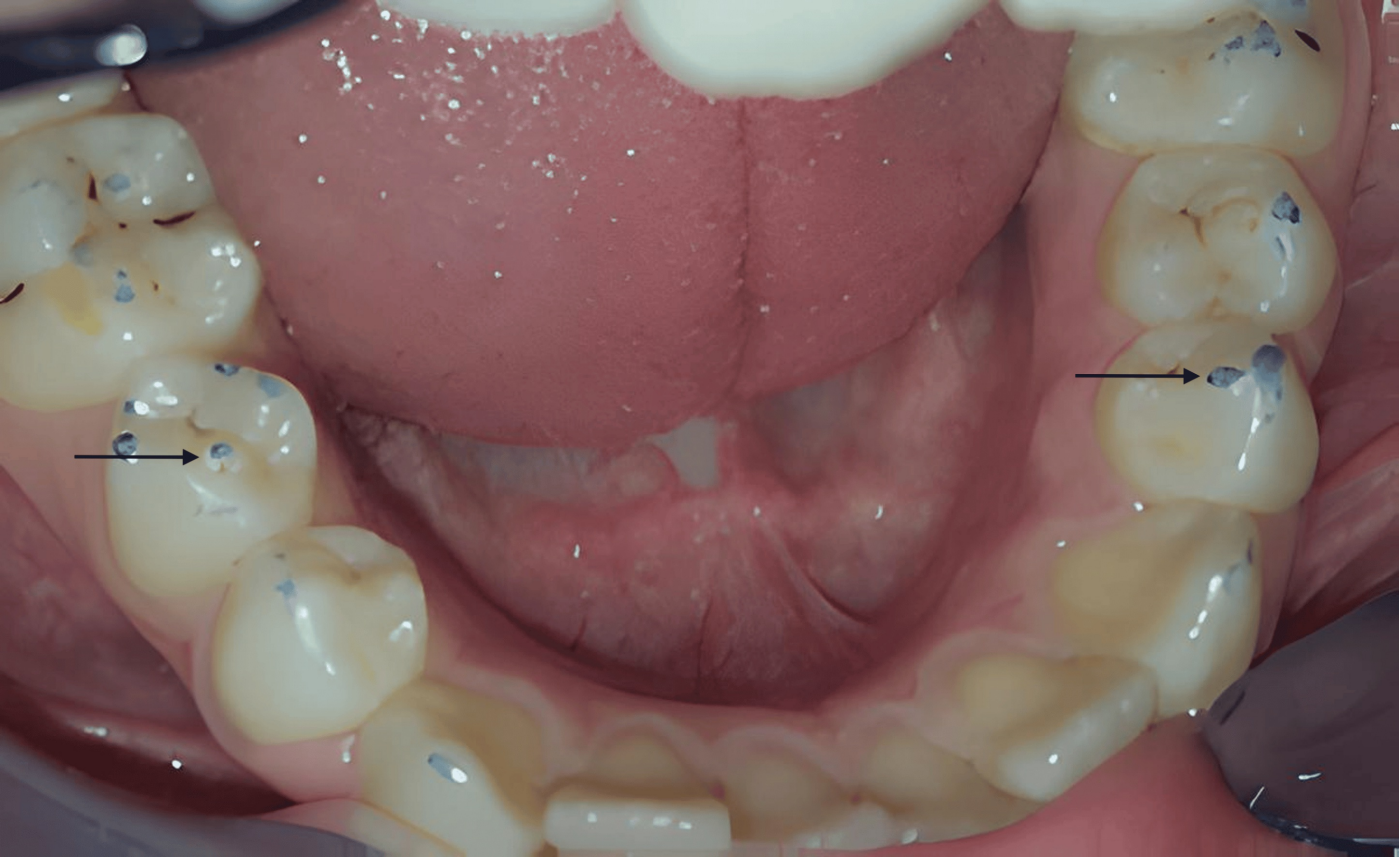
Control of the occlusion showed occlusal solicitations mostly with a tubercle on 45 and abrasions of both tubercles (black arrows)

Clinical examination indicated molar incisor hypomineralization (MIH) affecting the molars (Figure 1). The occlusal surfaces of the lower second premolars, 45 and 35, showed significant rounded projections of hard tissue located in the central grooves, between the buccal and lingual cusps (Figure 1). The lower left second premolar had a smaller projection at the crown's center. When occluded, the tubercle of tooth 35 did not make contact with the opposing teeth, while the tubercle of tooth 45 was in close occlusion with them (Figure 2).

**Figure 3:**
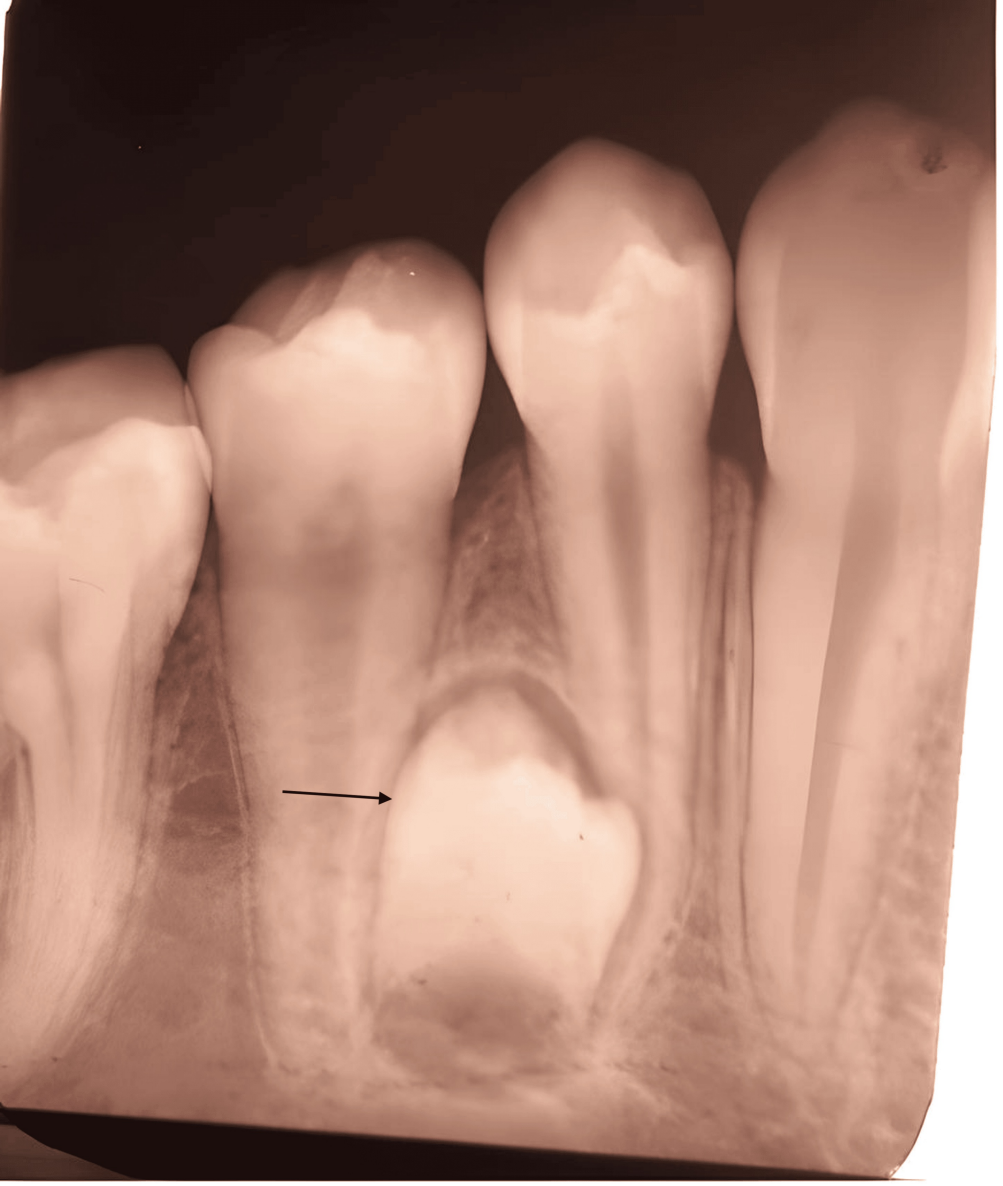
Intraoral periapical radiograph showing the absence of pulpal extensions into dens evaginatus and supernumerary premolars (black arrow) (right side)

**Figure 4:**
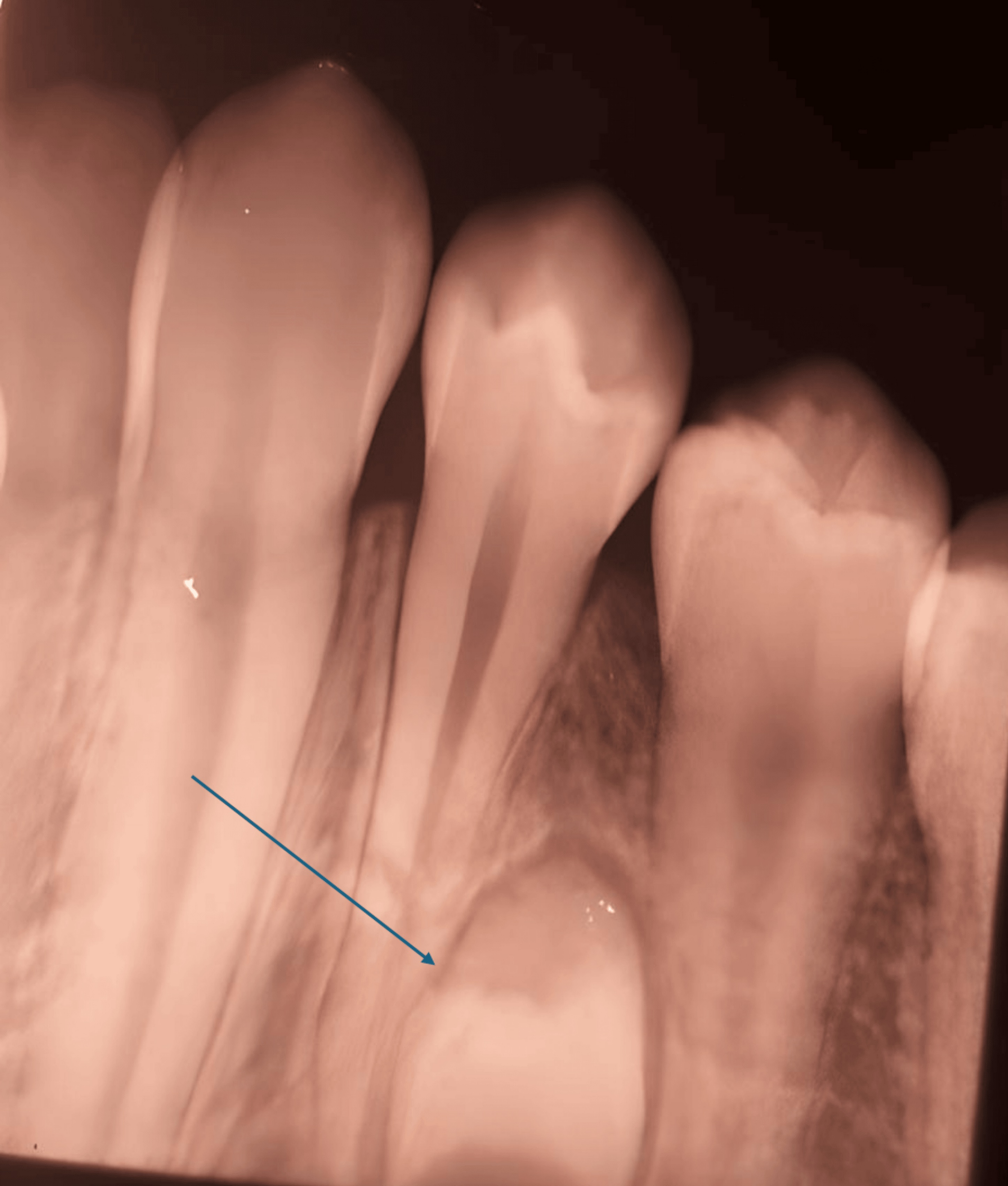
Intraoral periapical radiograph showing the absence of pulpal extensions into dens evaginatus and supernumerary premolars (black arrow) (left side)

Intraoral X-rays revealed no pulpal extensions into the dens evaginatus for teeth 45 and 35. Additionally, anomalous supernumerary teeth were visible in radiographic images between the roots of the lower left and right premolars (Figures 3, 4).

**Figure 5:**
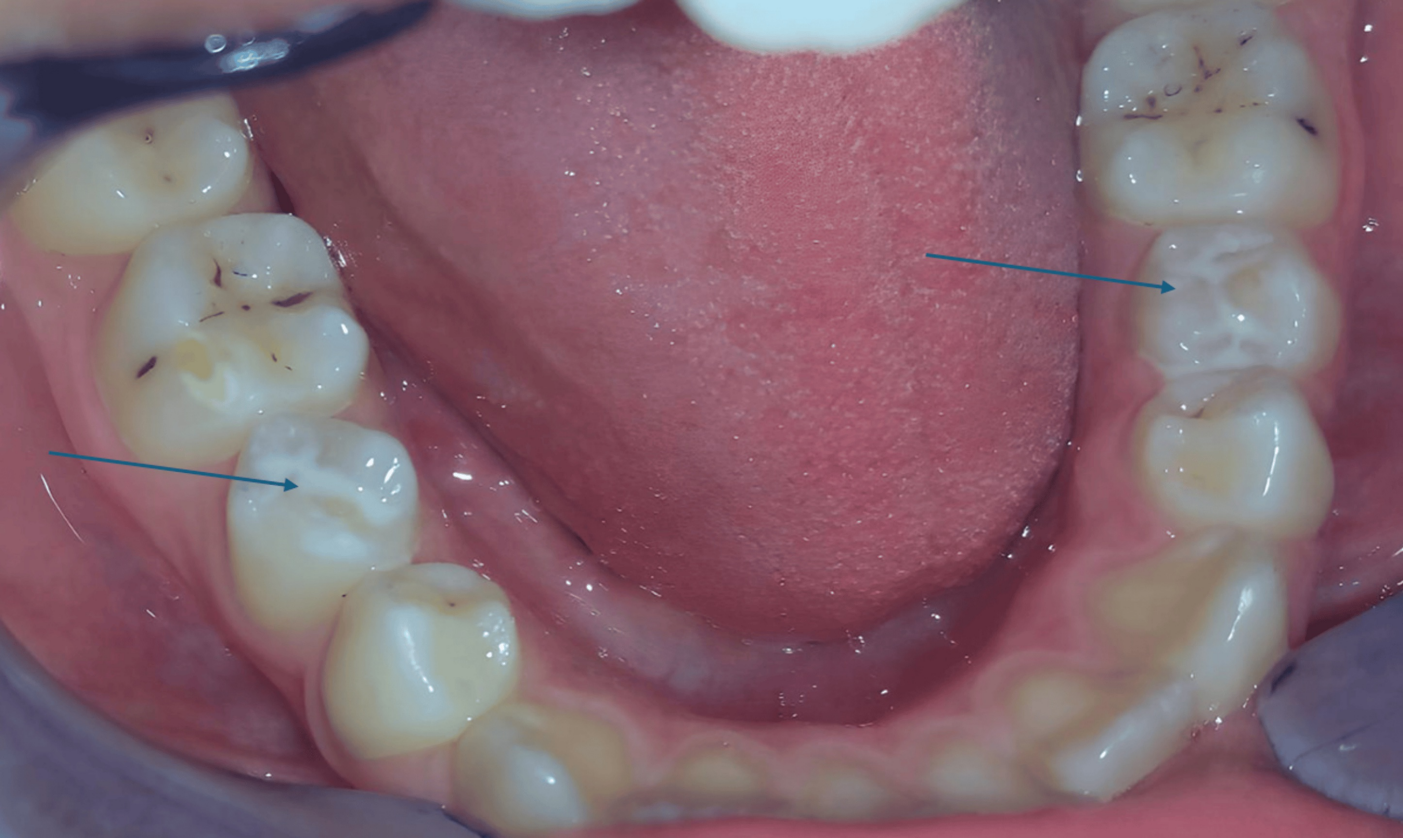
Final treatment with sealants after the slight reduction of tubercles on teeth 35 and 45 (blue arrows)

Hence, we determined that dens evaginatus was present on both teeth 45 and 35 without pulpal extensions. Given the lack of pulpal extension seen in the X-rays, this clinical situation permitted us to adopt a preventive treatment approach involving a slight reduction of the tubercles on teeth 45 and 35, followed by the application of sealants (light-cured resin-based fissure) to prevent hypersensitivity and caries (Figure 5).

Patient consent

Written informed consent was obtained from the patient and his legal guardian for the publication of this case report and accompanying images. All identifying information has been removed to ensure patient anonymity.

## Discussion

Dens evaginatus (DE), also referred to as tuberculum anomalum, is a developmental tooth anomaly characterized by an elevation of enamel, an abnormal tubercle, or a bulge, typically found in the main grooves of the molars and premolars. Mandibular premolars are the most frequently affected. DE can manifest on any tooth type, including the molars, canines, and incisors, with a higher occurrence in the mandible, five times more than in the maxilla, but it is primarily associated with the premolars [4,5].

This anomaly usually shows bilateral, symmetric distribution and a slight predisposition in women [1,3,6]. DE can affect both primary and permanent teeth, though it is most commonly found in permanent dentition [3]. Various dental anomalies, such as mesiodens, dens invaginatus, gemination, macrodontia, multiple unerupted teeth, and supernumerary mandibular premolars, have also been noted in patients with DE, as evidenced in this case [2,3].

The prevalence of this dental anomaly ranges from 1% to 4%, with higher rates observed among individuals of Mongoloid descent, highlighting significant racial differences. Reports indicate its occurrence in 3%-4.8% of Chinese and Eskimo populations, while it is relatively rare among White populations [1,2].

The precise etiology remains unclear; however, there is some evidence advocating for a familial or hereditary nature [2]. Several researchers suggest that hereditary factors might play a role, indicating a genetic component in its etiology [2,3]. The formation of the DE tubercle is a result of the proliferation and evagination of a segment of the inner enamel epithelium along with its underlying dental papilla into the stellate reticulum of the enamel organ [5,3]. This process leads to the formation of what is defined as a tubercle, characterized as a supplemental solid elevation in a specific area of the crown surface [2].

A tripartite classification for talon cusps was proposed by Hattab et al. (1996): type 1: talon, a well-defined additional cusp projecting prominently from the palatal or lingual surface and extending at least halfway from the cementoenamel junction (CEJ) to the incisal edge; type 2: semi-talon, an additional cusp of moderate size that extends less than halfway between the CEJ and the incisal edge; and type 3: trace talon, a prominent cingulum or a tubercle-like elevation, often conical, bifid, or in the form of an enlarged cingulum [6].

In our case, the DE in the mandibular right second premolar is a major DE, while on the contralateral side, it is a minor DE. The most common issue with an evaginated tooth is occlusal interference, which can lead to fractures or abrasion, resulting in pulpitis or pulpal necrosis and potentially periapical inflammatory lesions due to pulp irritation within the damaged tubercle. This condition is likely to occur in the early phases of root development, causing problems with normal growth. Maintaining cleanliness between the nodule and the tooth is difficult, and caries often occurs.

An unusual tubercle or elevation on the tooth surface is common, but damage may obscure the evidence of malformation. The awareness of dens evaginatus and its progression is crucial for timely diagnosis and treatment. Various treatment options for DE are reported in the literature, evolving over time. Most authors recommend prophylactic treatment of affected teeth soon after eruption.

Suggested prophylactic treatments include selective grinding, resin application, prophylactic restorations, and partial pulpotomy with mineral trioxide aggregate (MTA). Prophylactic interventions should occur shortly after eruption to avoid treatment for teeth with immature apices and weak roots. If pulp exposure is avoided after complete root formation, the tubercle remains prone to fracture [4,7].

If there is no occlusal interference, preventive measures based on fissure depth should be considered. Shallow fissures may receive fluoride, while deep fissures should be treated with sealants to prevent caries and reinforce the tubercle. If occlusal interference is present, DE management depends on pulp horn extension.

If the pulp horn does not extend into the tubercle, grinding, followed by composite restoration, is preferred. In this case, occlusal interferences exist without significant pulpal extensions or deep fissures, leading us to choose a double preventive approach: slight grinding of both tubercles, followed by sealant application. For mild pulp extension, progressive intermittent grinding is suggested to promote reparative dentin formation and pulpal recession, with fluoride application to reduce sensitivity [7].

If pulp exposure occurs, direct pulp capping is advised to maintain vitality. In cases of severe pulp extension, complete tubercle removal and associated pulpal procedures based on vitality and root development status are preferred. The extraction of DE teeth should be considered for orthodontic reasons or if apexification has failed or is contraindicated and also if the DE tooth is classified as a mesiodens or supernumerary tooth [8].

## Conclusions

This case emphasizes the significance of the early preventive management of dens evaginatus to safeguard pulp vitality and avoid long-term complications. Conservative treatment, consisting of slight tubercle reduction and sealant application, proved effective in managing bilateral DE without pulpal involvement.

The present report underlines the need for clinicians to remain vigilant in identifying this anomaly, to intervene promptly, and to follow patients longitudinally to ensure the stability and success of preventive measures.
